# A comparison of tests for Hardy-Weinberg Equilibrium in national genetic household surveys

**DOI:** 10.1186/1471-2156-14-14

**Published:** 2013-03-01

**Authors:** Yan Li

**Affiliations:** 1 Joint Program in Survey Methodology, University of Maryland at College Park, 7965 Baltimore Ave, College Park, MD, 20742, USA; 2 Department of Mathematics, University of Texas at Arlington, 701 S Nedderman Dr, Arlington, TX, 76019, USA

**Keywords:** Complex sampling, Condensed coefficients of identity, Quasi-score test, Taylor linearization

## Abstract

**Background:**

This study is motivated by National Household Surveys that collect genetic data, in which complex samples (e.g. stratified multistage cluster sample), partially from the same family, are selected. In addition to the differential selection probabilities of selecting households and persons within the sampled households, there are two levels of correlations of the collected genetic data in National Genetic Household Surveys (NGHS). The first level of correlation is induced by the hierarchical geographic clustered sampling of households and the second level of correlation is induced by biological inheritances from individuals sampled in the same household.

**Results:**

To test for Hardy-Weinberg Equilibrium (HWE) in NGHS, two test statistics, the CCS method [1] and the QS method [2], appear to be the only existing methods that take account of both correlations. In this paper, I evaluate both methods in terms of the test size and power under a variety of complex designs with different weighting schemes and varying magnitudes of the two correlation effects. Both methods are applied to a real data example from the Hispanic Health and Nutrition Examination Survey with simulated genotype data.

**Conclusions:**

The QS method maintains the nominal size well and consistently achieves higher power than the CCS method in testing HWE under a variety of sample designs, and therefore is recommended for testing HWE of genetic survey data with complex designs.

## Background

This study is motivated by population-based family data collected from National Genetic Household Surveys (NGHS), in which complex samples (i.e. sample collected with stratified multistage cluster sampling), partially from the same family, are selected. There are two levels of correlations of the collected genetic data in NGHS. The first level of correlation is induced by the hierarchical geographic clustered sampling of households and the second level of correlation is induced by biological inheritances from individuals sampled in the same household. Moreover, national household surveys often apply differential selection probabilities of selecting households and persons within the sampled households.

NGHS from various countries, such as the Canadian Health Measures Survey [3], Health 2000 Survey from Finland [4] and the US National Health and Nutrition Examinations Survey [5], have collected blood samples, from which DNA can be extracted for genetic analyses. For example, the NHANES III, a nationally representative survey of the U.S. population conducted by National Center for Health Statistics (NCHS), has genotyped candidate genes for participants 12 years and older. NHANES III employed a complex sample design, involving stratified multistage cluster sampling, to select participants [6,7]. Multiple blood-related individuals are often sampled from the same household. On average 1.6 persons are sampled per household [8].

There are at least three complications to analyze data collected in NGHS: 1) differential population weights, 2) hierarchical geographical correlation among families, and 3) genetic correlation within families. As known, genetic variability can differ by race [9] and social-economic factors [10]. The same factors, in complex sample designs, are often used to define sampling strata. Different selection probabilities are applied to each stratum and oversampling is often conducted to increase the efficiency of the estimates for certain subgroups (e.g. African Americans were oversampled in NHANES III). As a result, *differential population weights* related to genetic variability are associated with participants in the study. The second complication of *geographic correlation* in genetic variations is induced because race and social-economic status of individuals vary by location of residence, and multi-stage clustered sampling is often geography-based. Lastly, g*enetic correlation* is induced by genetic inheritance from biological parents within the household. Therefore, inflated variances of the estimates for the genetic quantities due to differential population weights, geographical intracluster correlation among households, and the genetic correlation within households are resulted. Consequently, the sample distribution with respect to genetic factors can be considerably different from the underlying population distribution. Researchers who implement complex sample designs should perform analyses with adjustments for the sample design complications. If analysis for simple random samples (SRS), instead, is performed on data collected with complex sample designs, the inference may be invalid.

Testing Hardy-Weinberg Equilibrium (HWE) of marker genotype frequencies has been widely recommended as a crucial step in genetic association studies [11-13]. The Hardy- Weinberg principal states that without disturbing genetic-related factors (e.g., non-random mating, selection, migration, or mutation) the genotype frequencies at an autosomal locus will attain equilibrium (i.e., HWE) in a single generation and maintain this equilibrium in future generations. Testing for HWE is useful because for non-codominant loci that are in HWE, genotype frequencies can be estimated from allele frequencies or vice versa, and more powerful genetic association studies are possible [14]. A variety of test methods have been developed to test HWE in SRS [15-20]. There are, however, limited literatures in developing HWE tests for genetic data collected in NGHS, considering both levels of correlations and differential weights.

Methods developed by She et al. [1] and Li et al. [2] appears to be the only two HWE tests available that consider all of the three complications. Interestingly, both methods are derived along different directions. In brief, tests developed by She et al. [1] are essentially corrected Pearson Chi-Square (CCS) tests. The exact joint distribution of the three genotypes from father, mother, and offspring were derived under HWE. Assuming a diallelic locus (i.e. alleles *A* and *a*) of autosomal genes, there are 10 possible genotype combinations among parents-child triads when ignoring the mating orders of father and mother. If HWE holds, the observed number of families in each of the 10 categories follows a multinomial distribution. Departure from HWE was then tested with the corrected Pearson chi-square statistics, including first-order correction, second-order correction, or a Satterthwaite F-version second-order correction [21,22]. By contrast, Li et al. [2] proposed a quasi-score (QS) test based on quasi-generalized estimating equations (GEE). Different from the regular GEE where the covariance matrix are often unknown, covariance matrix of the observed genotypes in the quasi-GEE are derived from the condensed coefficients of identity (CCI) [23], which appropriately measures the second level of genetic correlation among family members within families. The first level of correlation due to hierarchical geographic sampling of families is considered in the variance estimation of the estimated quasi- scores with respect to the fixation index (correlations between any pair of alleles within individuals, characterizing the departure from the HWE) using Taylor linearization methods.

In Li *et al*. [2], the QS method is claimed to advantage over the CCS method because the CCS method is limited to one type of family structure, i.e., 2 parents and 1 offspring (2P1O), while the QS method allows for a wide range of family structures. Trio (2P1O), however, is one of the common study designs in genetic association studies, e.g. transmission disequilibrium test. For the analysis of genetic data from 2P1O, Li et al. [2] didn’t make thorough comparisons between the two methods analytically or numerically. In addition, the QS and the CCS methods appear to be the only two existing methods that take account of the two levels of clustering effects induced by the national genetic household surveys (NGHS) (i.e. geographic correlation among families within PSUs and genetic correlation within families). Which method should be recommended in the analysis of NGHS genetic data collected from 2P1O families? For survey practitioners, it is important to select a proper test that maintains the nominal levels, but with higher power.

In this paper, we examine and compare the performance of two methods, in terms of the sizes and powers, via Monte-Carlo simulation studies under a variety of complex sample designs with differential weighting schemes and varying magnitudes of the correlation effects. It is observed that the QS method maintains the nominal size relatively well and consistently achieves higher power than the CCS method in testing HWE using data collected from 2 parents and 1 offspring in NGHS. In Section “Methods”, we outline the detailed methodology of the CCS and the QS tests. Both methods are compared in Section “Results” via simulation studies on the finite sample performance and applied to a real data example from the Hispanic Health and Nutrition Examination Survey with generated genotype data. Finally, the paper is wrapped up in Section “Conclusions”.

## Methods

Consider household surveys with stratified multistage cluster sample designs such as used in NHANES. These types of sample designs are described briefly as follows: The population of individuals is subdivided into disjoint primary sampling units (PSUs) usually based on the geographic locations of residence. For example, PSUs can be small cities or counties or contiguous cities/counties. The PSUs are grouped into strata so that they are approximately homogeneous with respect to certain demographic and geographic characteristics. At the first stage of sampling, a random sample of PSUs is selected from each stratum. At the second stage, smaller geographical units, so called secondary sampling units (SSUs), are randomly sampled from the sampled PSUs. Households/families are further randomly selected from the sampled SSUs, and at the ultimate stage individuals are randomly selected from sampled households/families. For each sampled individual, the inclusion probability is the product of the inclusion probabilities at each stage of sampling, and the corresponding sample weight is defined as the inverse of the inclusion probability. In most surveys the sample weights also involve adjustments for nonresponse and poststratification and can be considered as the number of people in the population represented by the sampled individual.

Let there be *H* strata with *I*_*h*_ PSUs in the *h*-th strata for *h* = 1, 2,…, *H*. Within the PSU-*hi* for *i* = 1, 2, …, *I*_*h*_, data is collected on *J*_*hi*_ families with *K*_*hij*_ individuals selected in the *j*th family and *j* = 1, 2, …, *J*_*hi*_.

### Corrected chi-square tests

She et al. [1] considered a diallelic locus, and the family structure of parents-child triads. The joint distribution of the three genotypes from the family under HWE was derived (see Table 1). Under simple random sampling setting, if HWE holds then the observed number of families in each of the ten categories has a multinomial distribution with parameter vector $\left(\overset{10}{\underset{l=1}{\sum }}J_{l},\pi \right),$ where *J*_*l*_ is the number of families in the *l*^th^ family type and $\pi ={\left(p_{A}^{4},2p_{A}^{3}p_{a},2p_{A}^{3}p_{a},2p_{A}^{2}p_{a}^{2},p_{A}^{2}p_{a}^{2},2p_{A}^{2}p_{a}^{2},p_{A}^{2}p_{a}^{2},2p_{A}p_{a}^{3},2p_{A}p_{a}^{3},p_{a}^{4}\right)}^{T}$ with *p*_*A*_ defined as the frequency of allele *A* and *p*_*a*_ the frequency of allele *a*.

**Table 1:** **Joint distributions of three genotypes from parents**-**child triads**

	**Genotype**			
**Familial type**	**Parents**	**Child**	**Joint probability**	**Count**
1	AA-AA	AA	$p_{A}^{4}$	*J*_1_
2	AA-Aa	AA	$2p_{A}^{3}p_{a}$	*J*_2_
3	AA-Aa	Aa	$2p_{A}^{3}p_{a}$	*J*_3_
4	AA-aa	Aa	$2p_{A}^{2}p_{a}^{2}$	*J*_4_
5	Aa-Aa	AA	$p_{A}^{2}p_{a}^{2}$	*J*_5_
6	Aa-Aa	Aa	$2p_{A}^{2}p_{a}^{2}$	*J*_6_
7	Aa-Aa	aa	$p_{A}^{2}p_{a}^{2}$	*J*_7_
8	Aa-aa	Aa	$2p_{A}p_{a}^{3}$	*J*_8_
9	Aa-aa	aa	$2p_{A}p_{a}^{3}$	*J*_9_
10	aa-aa	Aa	$p_{a}^{4}$	*J*_10_

The departure from HWE can be tested by Pearson chi-square test statistics [22]. Under complex sampling setting, each sampled individual represents certain number of persons (usually ≥ 1 depending on the sampling design) in the population. Accordingly, weighted version of Pearson chi-square test statistic was proposed and given by

(1) $$\chi_{A,W}^{2}=\theta {\left(J_{w},{\hat{\pi }}_{w}\right)}^{T}M\left(J_{w},{\hat{\pi }}_{w}\right)\theta \left(J_{w},{\hat{\pi }}_{w}\right),$$

where

*J*_*w*_ = (*J*_1*w*_, *J*_2*w*_, *J*_3*w*_, *J*_4*w*_, *J*_5*w*_, *J*_6*w*_, *J*_7*w*_, *J*_8*w*_, *J*_9*w*_, *J*_10*w*_)^*T*^ with *J*_*lw*_ representing the weighted number of families belonging to familial type *l* for *l* = 1, 2, …, 10; ${\hat{\pi }}_{w}$ is obtained by replacing allele frequencies *p*_*A*_ and *p*_*a*_  in ***π*** by ${\hat{p}}_{\mathrm{Aw}}=\left(4J_{1w}+3\left(J_{2w}+J_{3w}\right)+2\left(J_{4w}+J_{5w}+J_{6w}+J_{7w}\right)+J_{8w}+J_{9w}\right)/\left(4\overset{10}{\underset{l=1}{\sum }}J_{\mathrm{lw}}\right)$, and ${\hat{p}}_{\mathrm{aw}}=1-{\hat{p}}_{\mathrm{Aw}}$; $\theta \left(J_{w},{\hat{\pi }}_{w}\right)={\left(J_{w}-\left(\overset{10}{\underset{l=1}{\sum }}J_{\mathrm{lw}}\right){\hat{\pi }}_{w}\right)}^{T}$, i.e. the difference vector of observed number of families and the estimated expected number of families in each genotype combination; and

$$M\left(J_{w},{\hat{\pi }}_{w}\right)={\left(\overset{10}{\underset{l=1}{\sum }}J_{\mathrm{lw}}\right)}^{-1}{\left[\begin{array}{cccc} {\hat{p}}_{\mathrm{Aw}}^{4} & 0 & \cdots & 0\\ 0 & 2{\hat{p}}_{\mathrm{Aw}}^{3}{\hat{p}}_{\mathrm{aw}} & \cdots & 0\\ \vdots & \vdots & \ddots & \vdots \\ 0 & 0 & \cdots & {\hat{p}}_{\mathrm{aw}}^{4} \end{array}\right]}^{-1}$$

Due to both levels of correlations and the differential sample weights under the setting of complex survey design, the test statistics $\chi_{A,W}^{2}$ does not have asymptotic chi-square distribution. Therefore, She et al. [1] made several corrections based on the $\chi_{A,W}^{2}$ test. Via simulated and empirical studies, they recommended the use of the Rao-Scott first order corrected test in surveys like NHANES, and the test is given by

(2) $$\chi_{A,W}^{2}\left(1\right)=\chi_{A,W}^{2}/\hat{\bar{\lambda }},$$

where $\;\hat{\bar{\lambda }}=\sum_{i=1}^{8}{\hat{\lambda }}_{i}/8\;$ and $\;{\hat{\lambda }}_{i}\;$ are the non-zero eigenvalues of the matrix $\;\hat{\Sigma }M\left(J_{w},{\hat{\pi }}_{w}\right)$. Here $\hat{\Sigma }$ is a consistent estimator of covariance matrix of $\theta \left(J_{w},{\hat{\pi }}_{w}\right)$ derived by Taylor linearization method [24]. According to Rao and Scott [21], the $\chi_{A,W}^{2}\left(1\right)$ was asymptotically distributed as $\chi_{8}^{2}$ under H_0._ We also considered the following tests for comparison purpose.

(3) $$\chi_{A,W}^{2}\left(2\right)=X_{A,W}^{2}\left(1\right)/\left(1+{\hat{b}}^{2}\right),$$

where ${\hat{b}}^{2}=\frac{1}{8}\sum_{i=1}^{8}({\hat{\lambda }}_{i}-{\hat{\bar{\lambda }})}^{2}/{\hat{\bar{\lambda }}}^{2}$. The test $\chi_{A,W}^{2}\left(2\right)\;$ has an asymptotic chi-square distribution with degree of freedom (*df*) of 8/ $\left(1+{\hat{b}}^{2}\right)$ under the null hypothesis.

(4) $$F\chi_{A,W}^{2}\left(2\right)=\chi_{A,W}^{2}\left(2\right)/\left\{8\left(1+{\hat{b}}^{2}\right)\right\},$$

an F version of $\chi_{A,W}^{2}\left(2\right)$, is asymptotically distributed as F distribution with *df*_*numerator* = $8\left(1+{\hat{b}}^{2}\right)$ and $\mathrm{df}\_\mathrm{denominator}=\left(\sum_{h=1}^{H}I_{h}-H\right)$ under the null hypothesis.

### Quasi score test

Li et al. [2] suggested a quasi-score test for testing HWE. They considered a locus with *a* (≥2) different alleles and general familial relationships with family size *K*_*hij*_ (=1, 2, 3…). Define *M* = *a*(*a* + 1)/2, the number of possible distinct genotypes. The data are collected on the vector of variables **y**_***hijk***_ = ( *y*_*hijk.1*_,…, *y*_*hijk*.*g*_,…,*y*_*hijk*.*M*-*1*_) for each sampled individual, where *y*_*hijk*.*g*_ equals to 1 if individual-*hijk* has genotype *g* and 0 otherwise for *g* = 1, …, M-1. Define a parameter vector *θ* = (*p*, *r*)^*T*^, where ***p*** = (*p*_*1*_, …,*p*_*l*_, …, *p*_*a*-*1*_) denotes the (*a*-1) independent allele frequencies and the frequency of the last allele, $p_{a}=1-\sum_{1=1}^{a-1}pl$ and *r* denotes the fixation index. Under the null hypothesis of HWE, we have fixation coefficient *r* = 0.

Define E(**y**_***hijk***_) = **μ**_***hijk***_ with **μ**_***hijk***_ = ( *μ*_*hijk*.*1*_,…, *μ*_*hijk*.*g*_,…, *μ*_*hijk*.*M*-*1*_). If the genotype *g* is homozygote (e.g. *l*/*l*), $\mu_{\mathrm{hijk}.g}=\left(1-r\right)p_{l}^{2}+p_{l}$; if the genotype *g* is heterozygote (e.g. *l*/*l*' for allele *l* is not allele *l*'), *μ*_*hijk*. *g*_ = 2(1 − *r*)*p*_*l*_*p*_*l'*_. The estimating equations for the estimation of parameters *θ* are given by

(5) $$S\left(\theta \right)=\overset{H}{\underset{h=1}{\sum }}\overset{I_{h}}{\underset{i=1}{\sum }}\overset{J_{\mathrm{hi}}}{\underset{j=1}{\sum }}\frac{\partial \mu_{\mathrm{hij}}}{\partial \theta }W_{\mathrm{hij}}^{1/2}\mathrm{Va}r^{-1}\left(y_{\mathrm{hij}}\right)W_{\mathrm{hij}}^{1/2}\left(y_{\mathrm{hij}}-\mu_{\mathrm{hij}}\right)=0,$$

where ***y***_***hij***_ and ***u***_***hij***_ are vectors representing values over family members *k* = *1*,*2*…,*K*_*hij*_, and ***w***_***hij***_ represent a block-diagonal matrix whose block-matrices are *w*_*hij*_, *a* (*M*-*1*) *by* (*M*-*1*) diagonal matrix with each of diagonal elements the sampling weight associated to individual-*hijk*. Note that the quasi-estimating equation, constructed at the family level, considers genetic correlation among family members via ***Var***(***y***_***hij***_).

To simplify the notation, the subscript of the *j*th family in the *i*th PSU in the *h*th stratum (*hij*) is ignored. The covariance matrix ***Var***(***y***) for family-*hij* in (5) appropriately accounts for genetic correlation among family members within the family. For example, in the case of a biallelic locus (*A* and *a*) and the family structure of parents-child triads,

$$\mathrm{Var}\left(y\right)=\left[\begin{array}{ccc} \Sigma_{P} & \Sigma_{P,O_{1}} & \Sigma_{P,O_{2}}\\ & \Sigma_{O_{1}} & \Sigma_{O_{1},O_{2}}\\ \mathrm{SYS} & & \Sigma_{O_{2}} \end{array}\right],$$

where *SYS* denotes that ***Var***(***y***) is symmetric, and

$$\begin{array}{l} \Sigma_{P}=\Sigma_{O_{1}}=\Sigma_{O_{2}}=\left(\begin{array}{cc} p_{A}^{2}\left(1-p_{A}^{2}\right) & -2p_{A}^{3}p_{a}\\ \mathrm{SYS} & 2p_{A}p_{a}\left(1-2p_{A}p_{a}\right) \end{array}\right),\\ \Sigma_{P,O_{1}}=\Sigma_{P,O_{2}}=\left(\begin{array}{cc} p_{A}^{3}p_{a} & p_{A}^{2}p_{a}\left(1-2p_{A}\right)\\ \mathrm{SYS} & p_{A}^{2}p_{a}+p_{a}^{2}p_{A}-{\left(2p_{A}p_{a}\right)}^{2} \end{array}\right),and\\ \Sigma_{O_{1},O_{2}}=\left(\begin{array}{cc} \frac{1}{4}p_{A}^{2}+\frac{1}{2}p_{A}^{3}-\frac{3}{4}p_{A}^{4} & \frac{1}{2}p_{A}p_{a}-2p_{A}^{3}p_{a})\\ \mathrm{SYS} & p_{A}p_{a}\left(1-3p_{A}p_{a}\right) \end{array}\right). \end{array}$$

As known, family sizes and family relationships differ across families.

To test the null hypothesis of *r* = 0, a quasi-score test statistic was proposed. Let $\tilde{\theta }={\left({\tilde{p}}_{w},r=0\right)}^{T}$ denotes the solution to $S_{p}\left(\tilde{\theta }\right)=0,$, where ***S***_*p*_ is the first vector in the estimating Equations (5) and $S\left(\theta \right)={\left(S_{p}^{T},S_{r}^{T}\right)}^{T}$ is partitioned in the same way as *θ* Under suitable conditions [25,26], a quasi-score (*QS*) test statistic,

(6) $$\mathrm{QS}={\hat{S}}_{r}^{T}\left(\tilde{\theta }\right){\tilde{V}}_{L}^{-1}{\hat{S}}_{r}\left(\tilde{\theta }\right),$$

is asymptotically a *T*^*2*^ distributed variable with (*U*-*H*) denominator degrees of freedom with $U=\overset{H}{\underset{i=1}{\sum }}I_{h}$ the total number of sampled PSU’s, where ${\hat{V}}_{L}$ is a consistent estimator of the covariance matrix of ${\hat{S}}_{r}\left(\tilde{\theta }\right)$. Please refer to [2] for the estimation of covariance of ${\hat{S}}_{r}\left(\tilde{\theta }\right)$.

## Results

### Monte Carlo simulations

Let the finite population be of size *N* = 300,000 individuals consisting of M = 2,500 PSU’s with each PSU composed of 40 families with each family having 2 parents and 1 child. Considering a biallelic locus (allele *A* and allele *a*), the parental genotypes are generated independently according to a multinomial distribution with frequencies of $p\left(\mathrm{aa}\right)=\left(1-r\right)p_{\alpha }^{2}+r\;p_{\alpha }$, *p*(*Aa*) = 2(1 − *r*)*p*_*A*_*p*_*a*_, and $p\left(\mathrm{AA}\right)=\left(1-r\right)p_{A}^{2}+rp_{A}$. Given the parental genotypes, the genotype of the child is generated according to Mendelian law.

In the simulations, two different sample weight distributions are employed: (1) the sample weight value of one is assigned to all the sample individuals, i.e. *w* ≡ 1, so that there is no differential weighting effect; and (2) sample weight values of 1, 3, and 5 are each randomly assigned to one third of the sample of individuals, denoted by *w* ≡ {1, 3, 5}. The second weighting scheme is to mimic the real situation where the sample weights are noninformative of the genetic data. For example, surveys oversample the older person or younger children (e..g NHANES), leading to differential sample weights that are independent of the genotypes. The type I error rate and power are calculated based on 1,000 simulation runs with varying values of allele *A* frequency *p*_A_ = 0.1, 0.3, or 0.5. We calculate the rejection rates, defined by the proportion of 1,000 simulation runs for which the p-value is less than the significance level α (=0.05), to evaluate the performance of the five tests $\chi_{A,W}^{2},\chi_{A,W}^{2}\left(1\right),\chi_{A,W}^{2}\left(2\right),F\chi_{A,W}^{2}\left(2\right)$ and *QS*, given by (1), (2), (3), (4), and (6), respectively. Please recall the first 4 tests were proposed by She et al. [1] and the *QS* test was proposed by Li et al. [2].

In the first simulation study, the genetic data are correlated among family members, but independent among families within PSUs. Specifically, we select 60 PSU’s by simple random sampling from 2,500 PSU’s. As described above, the genetic information for each pair of parents of the 40 families in the PSU are independently generated by multinomial distributions. Thus, the genetic information among the families within PSUs is independent. Table 2 presents the sizes and powers given by the five tests when *p*_A_ = 0.3. The results when *p*_A_ = 0.1 or 0.5 showed the similar pattern, and therefore not shown. It can observed that the sizes of $\chi_{A,W}^{2}\left(1\right)\;$ and *QS* (when *r* = 0) maintain the nominal level across different weighting strategies (please see the bolded numbers in Table 1). Consistent with the findings in [1], the $\chi_{A,W}^{2}\left(2\right)$ and $F\chi_{A,W}^{2}\left(2\right)$ are conservative, especially under the case of differential weighting strategy *w* ≡ {1, 3, 5}. When *r* = 0.03, the powers achieved by the $\chi_{A,W}^{2}\left(1\right)$ is slightly higher than the $\chi_{A,W}^{2}\left(2\right)$; whereas the *QS* test achieves the greatest power, more than 50 % higher than the power by $\chi_{A,W}^{2}\left(1\right)$. For example, under *w* ≡ {1, 3, 5} the power of *QS* test is 66 % [=(0.209-0.126)/0.126] higher than the power achieved by the $\chi_{A,W}^{2}\left(1\right)$ test. The similar pattern can be observed when *r* = 0.05.

**Table 2:** **Sizes and powers given by five HWE tests**: **40 families within each PSU are independent**

	^#^***r*** = **0**		***r*** = **0**.**03**		***r*** = **0**.**05**	
*w*	≡1	≡{1,3,5}	≡1	≡{1,3,5}	≡1	≡{1,3,5}
$\chi_{A,W}^{2}{}^{*}\;$	**0**.**056**	0.856	0.178	0.932	0.667	0.994
$\chi_{A,W}^{2}{\left(1\right)}^{*}\;$	**0**.**049**	**0**.**044**	0.163	0.126	0.646	0.512
$\chi_{A,W}^{2}{\left(2\right)}^{*}\;$	**0**.**038**	0.032	0.140	0.106	0.616	0.478
$F\chi_{A,W}^{2}{\left(2\right)}^{*}\;$	0.030	0.021	0.103	0.085	0.551	0.408
QS^**^	**0**.**043**	**0**.**047**	0.244	0.209	0.800	0.697

^*^ Tests proposed by She et al. (2009).

^**^Tests proposed by Li et al. (2011).

^#^Sizes ranging from 0.036 $\left(=0.05-1.96\times \sqrt{.05\times .95/1000}\right)$ to 0.064 $\left(=0.05+1.96\times \sqrt{.05\times .95/1000}\right)$ maintain the nominal level and are bolded.

In the second simulation study, the genetic data are correlated among family members as well as among families within PSUs. In order to introduce the correlation among families within PSUs, we generate a clustered finite population. In detail, we sort all the 100,000 families by the number of genotype *aa* within the family. The 2,500 PSU’s are then formed by grouping every 40 families sequentially in the sorted population. Thus, the genetic information among the families in each PSU is correlated. A simple random sample of 60 PSU’s is then selected from the 2,500 reformed PSU’s. Table 3 presents the sizes and the powers of the five HWE tests when the families in each PSU are correlated. As expected, the sizes are conservative for $\chi_{A,W}^{2}\left(2\right)$ and $F\chi_{A,W}^{2}\left(2\right)$ tests. The $\chi_{A,W}^{2}\left(1\right)$ test, however, produces inflated sizes ranging from 0.11 ~ 0.12. The QS test maintains the nominal size relatively well. In terms of the power, however, the *QS* test consistently achieves higher power than $\chi_{A,W}^{2}\left(1\right)$, $\chi_{A,W}^{2}\left(2\right)$ or $\;F\chi_{A,W}^{2}\left(2\right)$ when fixation coefficient *r* = 0.1 or 0.2 across different weighting strategies.

**Table 3:** **Sizes and powers given by five HWE tests**: **40 families within each PSU are correlated**

	^#^***r*** = **0**		***r*** = **0**.**1**		***r*** = **0**.**2**	
***w***	≡**1**	≡{**1**,**3**,**5**}	≡**1**	≡{**1**,**3**,**5**}	≡**1**	≡{**1**,**3**,**5**}
$\chi_{A,W}^{2}{}^{*}\;$	0.998	1.000	1.000	1.000	1.000	1.000
$\chi_{A,W}^{2}{\left(1\right)}^{*}\;$	0.116	0.111	0.215	0.210	0.539	0.535
$\chi_{A,W}^{2}{\left(2\right)}^{*}\;$	0.035	0.031	0.078	0.072	0.242	0.232
$F\chi_{A,W}^{2}{\left(2\right)}^{*}\;$	0.027	0.024	0.065	0.061	0.218	0.204
QS^**^	0.076	0.071	0.215	0.216	0.668	0.667

^*^Tests proposed by She et al. (2009).

^**^Tests proposed by Li et al. (2011).

^#^Sizes ranging from 0.036 $\left(=0.05-1.96\times \sqrt{.05\times .95/1000}\right)$ to 0.064 $\left(=0.05+1.96\times \sqrt{.05\times .95/1000}\right)$ maintain the nominal level and are bolded.

By comparing results from two simulation studies (see Tables 2 and 3), it can be observed that the tests are more powerful when the families within PSUs are independent, relative to that when the families within PSUs are correlated. For example, under *w* ≡ {1, 3, 5}, it requires *r* = 0.03 for the *QS* test to achieve the power of 0.209 when the families are independent (see last row of Table 2); whereas *r* needs to be as large as 0.10 for the *QS* to reach the similar power of 0.216 when there exists correlation within PSUs (see last row of Table 3).

In conclusion, the *QS* test appropriately incorporates two levels of correlations of genetic data, and thus maintain the nominal levels relatively well, and consistently achieve the highest power compared to $\chi_{A,W}^{2}\left(1\right)$, $\chi_{A,W}^{2}\left(2\right)$ or $\;F\chi_{A,W}^{2}\left(2\right)$, across different sampling strategies.

### Example from the Hispanic health and nutrition examination survey with generated genotype data

We use data from the Mexican-American part of the HHANES [27] that consisted of household interviews and physical examinations conducted between 1982 and 1984 for a random sample of non-institutionalized Mexican Americans aged 6 month to 74 years residing in selected areas in the southwestern part of the US.

The HHANES had a stratified multistage cluster sample design; see [27] for further details about the sample design. At the last stage of sampling, individuals were sampled from selected households with rates based on age: 50 % for 2–19 years, 75 % for 20–44 years, and 100 % for 45–74 years. This within-household sampling was accomplished systematically from a household roster obtained by the interviewer. Within a sampled household, all household members related by blood, marriage, or adoption were considered to be a family. To compare the *CCS* and *QS* methods, we restricted our analyses to families with two parents and one child, resulting in 307 sampled families. Thus, totally 921 sampled individuals are involved in the data analysis.

Since the HHANES did not genotype their sampled individuals, we generated genotype data using a two-step procedure by following [2]. In step 1, the allele *A* frequency *p*_A_ was generated from the Beta distribution *p*_*A*_ ~ Beta((1 − *r*)*f*_*A*_/ *r*, (1− *r*)(1 − *f*_*A*_)/ *r*) when *r* ≠ 0. When *r* = 0, *p*_*A*_ takes *f*_*A*_, where *r* is the fixation coefficient. In step 2, for each parent, two alleles were drawn at random from the binomial distribution Bin (2, *p*_*A*_). Given parental genotypes, the genotype of a child was randomly generated by the Mendelian law.

According to the within-family sampling design of HHANES, we set the within-family weights to be 2 if the individual is 2–19 years; 1.33 if the person is between 20–44 years; and 1 if the person is 45–74 years. The final sample weight for each sampled individual was provided and calculated by the product of inclusion probabilities at each stage of sampling with nonresponse and postratification adjustments. For construction of family-level weights, we follow [28] by taking the average of the remaining weights (= final sample weight / within-family weight) of 2 parents and 1 child in each of the sampled families. Family-level weights will be employed in the analysis.

We varied the values of the fixation coefficient *r* to be 0, 0.1, 0.2 and 0.3. Table 4 presents the p-values of $\chi_{A,W}^{2}\left(1\right)$, $\chi_{A,W}^{2}\left(2\right)$, $\;F\chi_{A,W}^{2}\left(2\right)$, and *QS* for testing Hardy-Weinberg Equilibrium (HWE) with specified *f*_*A*_ = 0.3. All the four tests take account of the correlation induced from the selection of the families, and the biological correlation within the family. Consistent with results from the simulation studies, *f*_*A*_ are conservative, producing larger p-values than $\chi_{A,W}^{2}\left(1\right)$ across varying values of *r* and *w*. All the four tests accept HWE when *r* = 0. The *QS* starts rejecting null hypothesis of HWE with p-values < 0.05 when *r* ≥ 0.1; whereas $\chi_{A,W}^{2}\left(1\right)$ accepts the null hypothesis of HWE when *r* = 0.1 with p-value = 0.207.

**Table 4:** P-value from Hispanic Health and Nutrition Examination Survey data analysis where the genotype data is generated with allele frequency *f*_A_ =0.3 and varying fixation coefficient *r*

	***r*** = **0**	***r*** =**0**.**1**	***r*** =**0**.**2**	***r*** =**0**.**3**
$\chi_{A,W}^{2}{\left(1\right)}^{*}\;$	0.347	0.207	**0**.**000**	**0**.**000**
$\chi_{A,W}^{2}{\left(2\right)}^{*}\;$	0.347	0.241	**0**.**006**	**0**.**000**
$F\chi_{A,W}^{2}{\left(2\right)}^{*}\;$	0.414	0.329	0.051	**0**.**013**
QS^**^	0.535	**0**.**014**	**0**.**000**	**0**.**000**

^*^Tests proposed by She et al. (2009); ^**^Tests proposed by Li et al. (2011).

## Conclusions

In this paper, we compared test statistics recently proposed by She et al. [1] and Li et al. [2] for testing Hardy-Weinberg Equilibrium of genetic data collected from 2P1O families in NGHS. Both methods consider two levels of clustering (correlation) effects with the first level induced by the hierarchical clustered sampling of families and the second level induced by the biological inheritance within families. Based on results from our small sample simulation studies, we have the following observations. *First*, when genotypes among families within PSUs are independent, i.e. there is no first-level correlation from sampling families, the sizes of $\chi_{A,W}^{2}\left(1\right)\;$ and *QS* (when *r* = 0) maintain the nominal level across different weighting strategies and the *QS* test achieves the greater power under alternative hypothesis of *r* > 0. *Second*, when genotypes among families within PSUs are correlated, the sizes are conservative for $\chi_{A,W}^{2}\left(2\right)$ and $F\chi_{A,W}^{2}\left(2\right)$ tests. Compared to $\chi_{A,W}^{2}\left(1\right)$, the *QS* test maintains the nominal level relatively well. In terms of the power, however, the *QS* test consistently achieves higher power than $\chi_{A,W}^{2}\left(1\right)$, $\chi_{A,W}^{2}\left(2\right)$ or $\;F\chi_{A,W}^{2}\left(2\right)$ across different sampling strategies. Therefore, based on our limited simulation results, we would recommend the *QS* test for testing HWE of NGHS genetic data.

## Discussion

We originally planned for testing HWE using the National Health and Nutrition Examination Survey III (NHANES III) genetic data. However, in order to access NHANES III genetic data, researchers are required to be onsite at NCHS in Hyattsville, Maryland. Being located out of the state of Maryland, we are not able to access the data. Considering the similar survey components in Hispanic Health and Nutrition Examination Survey (HHANES) as in the NHANES III, we decided to apply the developed tests to HHANES with simulated genotype data. Although the genetic data are simulated, all the sampling components, such as the stratification, hierarchical clustering, family size, and family relationships, are real, and thus the analysis can still serve as a useful illustration for testing HWE in NGHS. However, we admit that the fact of simulated genotypes in the real data analysis is one of the limitations in this study.

In the simulation studies, the family members are selected by family relationship (i.e. 2P1O). In real surveys, however, individuals could be selected by their phenotypic characteristics, e.g., diseased or disease-free, which are often correlated within certain susceptible genetic variations. The magnitude of this correlation will differ depending on the susceptible genetic variations of interest. In our simulation studies (results not shown), both methods produced biased estimates of allele frequencies and the type I error rate is inflated when within-family selection is highly related to the genotypes. In future research, an extension of the QS estimator will be studied to account for within-family weights that are correlated with genetic variations.

## Competing interests

The author declares that she has no competing interests.

## Authors’ contributions

YL designed overall study including designing the sampling schemes, implementing the simulation studies, performing the analysis of HHANES with generated genotypes, and writing the manuscript.
